# Effects of Subchronic Exposure to Cadmium and Diazinon on Testis and Epididymis in Rats

**DOI:** 10.1155/2014/632581

**Published:** 2014-12-07

**Authors:** Maria Adamkovicova, Robert Toman, Michal Cabaj, Peter Massanyi, Monika Martiniakova, Radoslav Omelka, Vladimira Krajcovicova, Hana Duranova

**Affiliations:** ^1^Department of Botany and Genetics, Constantine the Philosopher University, Nitra 949 74, Slovakia; ^2^Department of Veterinary Disciplines, Slovak University of Agriculture, Nitra 949 76, Slovakia; ^3^Department of Animal Physiology, Slovak University of Agriculture, Nitra 949 76, Slovakia; ^4^Department of Zoology and Anthropology, Constantine the Philosopher University, Nitra 949 74, Slovakia

## Abstract

The present study aimed to elucidate the structural changes in testis and epididymis of adult rats following subchronic peroral administration of cadmium at 30 mg/L, diazinon at 40 mg/L, cadmium at 30 mg/L, and diazinon at 40 mg/L, respectively. At the end of 90-day experiment, the samples of the testes and epididymis were assayed by qualitative and quantitative histological methods. The testis and epididymis weights increased following exposure to cadmium and simultaneous exposure to cadmium and diazinon. Testicular damage following cadmium and diazinon coexposure was significantly less expressive than in groups with individual administration of these compounds. Cadmium caused a significant thickening of seminiferous epithelium, cellular degeneration, and necrosis. Desquamation of immature germ cells resulted in a significant increase of intraepithelial spaces and reduced tubule volume in all experimental groups. Vascular dilation and congestion were detected in the interstitial tissue. The changes in epididymal histology in the group exposed to cadmium and group exposed simultaneously included a reduction of epithelium, necrotic epithelial cells, vasoconstriction, and interstitial edema together with mononuclear cell infiltration. Results did not indicate a synergistic or any additional effect from the simultaneous administration of both toxicants. Further research is needed to determine the significance and the mechanism of the adverse effects.

## 1. Introduction

The ability of chemical pollutants to affect reproductive health has garnered significant attention in recent decades and is further compounded by the accumulation of endocrine disruptive chemicals in the environment [1, 2]. Chemicals that disrupt normal endocrine function may interfere with the hormonal pathways responsible for the control of reproduction [3] triggering morphological and functional abnormalities [4, 5].

Cadmium is a ubiquitous environmental contaminant arising primarily from electroplating, plastics manufacturing, mining, paint pigments, alloy preparation, and batteries. Food is the most important source of cadmium in the nonsmoking, nonoccupationally exposed population [6]. Cadmium can produce both carcinogenic and noncarcinogenic effects on various organs including the lung, liver, kidney, bone, and vascular system [7]. At the cellular level, cadmium induces oxidative stress, cell proliferation, and apoptosis [8, 9]. Cadmium is a known endocrine disruptor and reproductive toxicant [10, 11] which affects male fertility through altered hypothalamic-pituitary-testicular axis function [12] and/or through direct gonadotoxic and spermiotoxic effects [13]. The disruption of junctional structures within the blood-testis [14, 15] and blood-epididymis barrier [16, 17] results in impaired spermatogenesis and sperm maturation processes associated with infertility [18].

The organophosphate insecticide diazinon is commonly used in agriculture and households to control pest insects in soil, plants, fruit, and vegetable crops [19]. Diazinon acts on the nervous system through the inhibition of the acetylcholinesterase activity at the synapses and neuromuscular junctions and is manifested by overstimulation of acetylcholine receptors and impeded neurotransmission [20]. The ubiquitous distribution of both nicotinic and muscarinic cholinergic receptors together with induction of oxidative stress in various tissues [21] may have genotoxic, immunotoxic, nephrotoxic, hepatotoxic, and cardiotoxic effects [22]. Diazinon suppresses reproductive function with endogenous hormonal disruption [5, 23] inducing histopathological alterations in testes and spermatogenic disturbances [24–26]. Exposure to diazinon negatively affects sperm motility and DNA integrity, which may contribute to reduced semen quality and concomitant decreases in fertility [27–29].

Humans are exposed to mixtures of chemicals which can interact. The most common approach to describe the combined action of the components in the mixture is to perform experimental studies comparing the effects of the mixture to the effects of the individual compounds [30, 31]. Therefore, the present study was aimed at identifying the possible interactions among cadmium and diazinon on testis and epididymis following subchronic peroral administration to rats because of their possible occurrence in the food chain.

## 2. Material and Methods

### 2.1. Chemicals

Cadmium in the form of cadmium chloride dehydrate compound (CdCl_2_ *·*2H_2_O), with purity 96%, was purchased from Reachem, Slovak Republic. Diazinon PESTANAL, analytical standard (C_12_H_21_N_2_O_3_PS), with purity 99%, was obtained from Sigma-Aldrich Laborchemikalien GmbH, Germany.

### 2.2. Animals

The male Wistar rats were individually housed in plastic cages in an environment maintained at 20–24°C, 55 ± 10% humidity, and 12/12 h cycle of light and darkness with access to food (feed mixture M3, Machal, Czech Republic) and drinking water* ad libitum*. All experiments were conducted in accordance with standard guide for the care and use of laboratory animals in an accredited laboratory (SK PC 50004, SUA Nitra).

### 2.3. Experimental Design

The 4-week-old rats were randomly assigned into 4 groups of 10 males each. Rats in group B were dosed with cadmium at 30 mg/L in drinking water for 90 days. Rats in group C were exposed to diazinon at 40 mg/L in drinking water for 90 days. Rats in the group D were given a mixture of cadmium and diazinon (30 mg/L and 40 mg/L, resp.) in drinking water for 90 days. All experimental groups were compared to a control group A with no intervention. The dose regimen and route of administration were chosen based on the experimental precedents set in the literature in order to produce a target organ toxicity arising from repeated exposure which does not induce animal mortality [32, 33]. The rats were observed daily for survival and clinical signs of toxicity. Individual body weights, food consumption, and water consumption were measured at weekly intervals.

### 2.4. Histological Study

Ninety days after starting the experiment, the animals were sacrificed while still under anesthesia by cervical dislocation and given a limited gross necropsy with a focus on the reproductive organs. The testes and epididymis were removed from the scrotum, freed from adherent tissues, weighed on analytical scales (JL-180, Chyo Balance Corp., Japan), and fixed in modified Davidson's solution [34]. Each portion was dehydrated in a graded series of ethanol, saturated in benzene, benzene-paraffin, embedded in paraffin wax, sectioned at 5 *μ*m, and stained with hematoxylin-eosin [35]. Ten sections per organ of each animal were randomly chosen for light microscopy evaluation (Nikon Eclipse E600, Kawasaki, Kanagawa, Japan) at 200x magnification to detect qualitative histological changes produced by cadmium and diazinon.

### 2.5. Morphometric Analysis

Morphometric methods [16, 36] were used to quantify the structural parameters of individual tissue in ten fields per section based on computerized techniques with PC morphometric software M.I.S. QuickPhoto associated with light microscope Olympus AX 70 Provis (Olympus, Tokyo, Japan) at 400x magnification. The following measurements were performed in testis tissue: relative volume and surface area of seminiferous epithelium, intraepithelial spaces, tubule lumen, blood vessels, interstitium, tubule surface area, and tubule diameter. The following measurements were performed in epididymis tissue: relative volume and surface area of tubule epithelium, tubule lumen, blood vessels, interstitium, tubule surface area, and tubule diameter.

### 2.6. Statistical Analysis

Data was expressed as the mean ± standard deviation. Differences were tested for statistical significance by one-way analysis of variance (ANOVA) and* post hoc* Scheffe's test using SAS 9.2 Enterprise Guide 4.3 software (SAS Institute Inc., Cary, NC, USA). The significance level was set at 5% (^*^ *P* < 0.05, ^**^ *P* < 0.01, and ^***^ *P* < 0.001).

## 3. Results

### 3.1. Biometry and Morphometry

Weekly body masses and food intake were not significantly different among the groups (data not shown). There were also no significant differences in final body weights. The cadmium administration significantly increased the testis (*P* < 0.05) and epididymis weight (*P* < 0.01). Furthermore, significant increases in weight of testes (*P* < 0.01) and epididymis (*P* < 0.001) following simultaneous exposure to cadmium and diazinon were detected. The biometric data is presented in Table 1.

Relative volume and surface areas of testicular tissue components were calculated. Morphometric analysis demonstrated a significant reduction (*P* < 0.001) of the seminiferous epithelium in the group exposed to cadmium accompanied by a significant increase of intraepithelial empty spaces (*P* < 0.001) in all experimental groups. Tubule lumen significantly decreased in all experimental groups, exposure to cadmium (*P* < 0.05), exposure to diazinon (*P* < 0.001), and simultaneous exposure to cadmium and diazinon (*P* < 0.01). The relative volume of interstitial tissue did not differ significantly between the groups. Vascular surface area and vascular volume increased significantly in the cadmium group (*P* < 0.001), the diazinon group (*P* < 0.001), and the group with combined exposure (*P* < 0.01). No alteration was detected in the surface area and diameter of the seminiferous tubule.

Volume fractions and surface areas of epididymal tissue components were calculated. Epithelium was significantly reduced in the group exposed to cadmium (*P* < 0.01) and in the group exposed simultaneously (*P* < 0.05). A significant lumen extension in the cadmium exposed group was detected (*P* < 0.05). The combined exposure group exhibited significantly widened interstitial tissue (*P* < 0.001). Subchronic cadmium and combined exposure induced significant vascular constriction (*P* < 0.05). The significantly increased tubule surface area (*P* < 0.05) corresponds with significantly distended diameter of the epididymal tubule in the group exposed to cadmium. The morphometric data is summarized in Tables 2 and 3.

### 3.2. Histopathology

**Figure 1:**
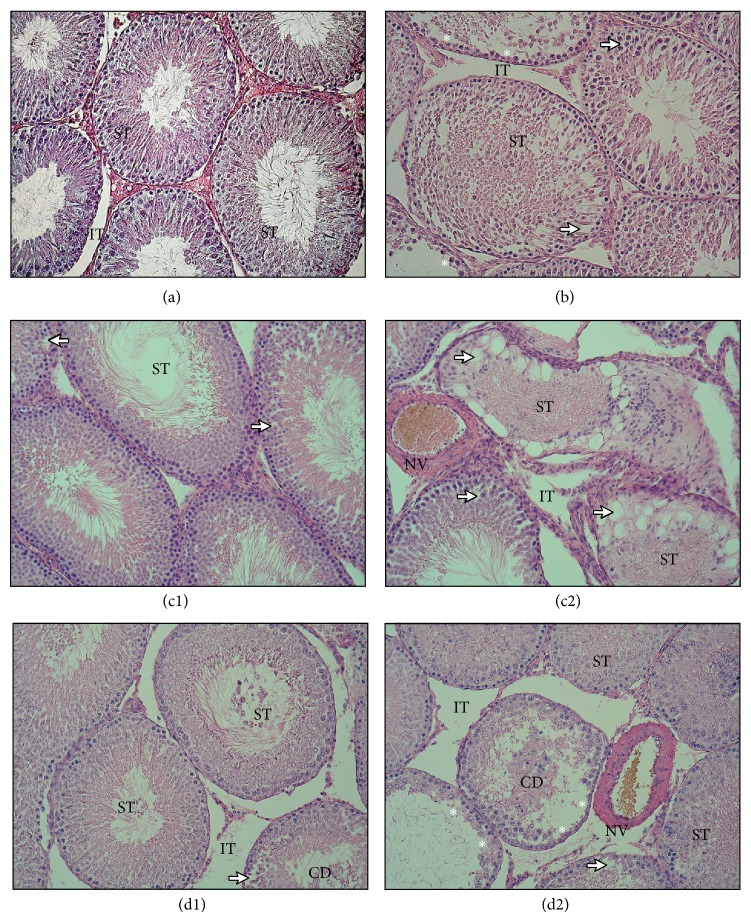
Photomicrographs showing histology of testicular tissue from control group (a), group exposed to cadmium at a dose of 30 mg/L (b), group exposed to diazinon at a dose of 40 mg/L (c1, c2), and group exposed to cadmium at a dose of 30 mg/L and diazinon at a dose of 40 mg/L (d1, d2). ST: seminiferous tubules, IT: interstitial tissue, CD: cellular debris, NV: necrotizing vasculitis, arrows: intraepithelial empty spaces, and stars: seminiferous tubules lined only by Sertoli cells (hematoxylin-eosin, original magnification ×200).

In the control group, testes showed normal testicular architecture. Well-developed circular or elliptical seminiferous tubules were enclosed by a thick basal lamina. The tubules were lined with seminiferous epithelium with active spermatogenesis. The germ cells were organized into concentric layers in close contact with Sertoli cells. Interstitial tissue was filled with interstitial Leydig cells, small blood vessels, collagen fibres, and fibroblasts (Figure 1(a)). Cadmium subchronic exposure resulted in moderate to severe testicular degeneration and distortion accompanied by lumen contraction (Figure 1(b)). While interstitial tissues were generally unaffected, blood vessels appeared dilated and more congested. Reduced seminiferous epithelium showed desquamation of immature germ cells with vacuole formation. Nevertheless, many seminiferous tubules showed germ cell disorganization with necrotic cellular debris, and numerous tubular lumens contained a number of mature sperms. It was found in the testis of rats exposed to diazinon that most tubules did not corroborate a significant change of germinal epithelium and interstitial connective tissue (Figure 1(c1)); however, some others appeared markedly necrotic, with degeneration of epithelial cells and only remnants of the basement membrane (Figure 1(c2)). The altered seminiferous tubules showed irregular shape, disarranged epithelial layers, and lumen filled with detached germ cells. In the most severely damaged tubules, the germ cells were not detectable, and several multinucleated bodies and cells were frequently found together with large vacuoles. Rats receiving cadmium and diazinon simultaneously showed both normal (Figure 1(d1)) and damaged seminiferous tubules in testis. The damaged tubules exhibited disorganization and degeneration of seminiferous epithelium. In particular, they lacked the characteristic basal to luminal maturation of germ cells, which was induced by desquamation of cells into the tubule lumen creating the intraepithelial empty spaces (Figure 1(d2)).

**Figure 2:**
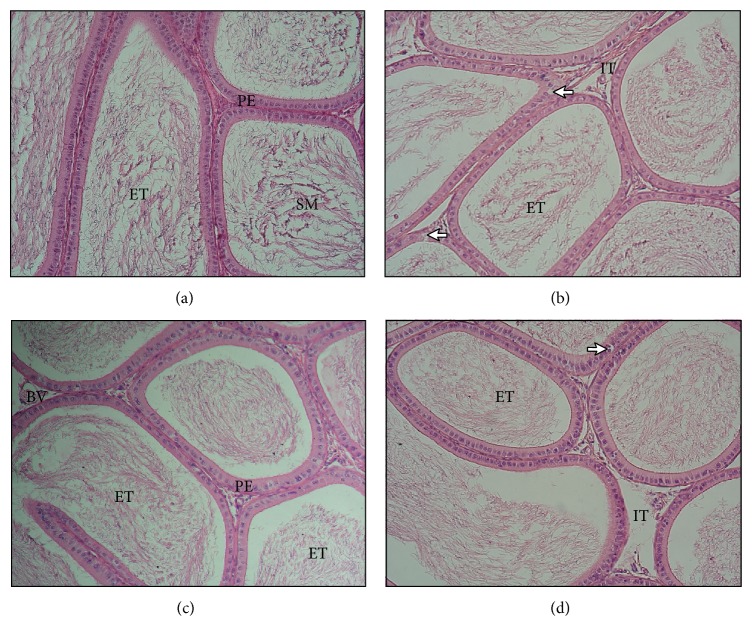
Photomicrographs showing histology of epididymal tissue from control group (a), group exposed to cadmium at a dose of 30 mg/L (b), group exposed to diazinon at a dose of 40 mg/L (c), and group exposed to cadmium at a dose of 30 mg/L and diazinon at a dose of 40 mg/L (d). ET: epididymal tubules, PE: pseudostratified columnar epithelium, IT: interstitial tissue, SM: spermatozoa, BV: blood vessels, CD: cellular debris, and arrows: intraepithelial empty spaces (hematoxylin-eosin, original magnification ×200).

The cross sections of cauda epididymis obtained from the control group revealed regular and circular tubules with a pseudostratified columnar epithelium that exhibits stereocilia. A dense collection of sperm cells in the tubular lumen was evident. Extratubular space contained blood vessels in the interstitial connective tissue. The epithelium was separated from the connective tissue by an intact basement membrane (Figure 2(a)). Histological analysis did not reveal differences in epididymal morphology of rats, except for the vacuole formation within pseudostratified epithelium following cadmium (Figure 2(b)) and combined cadmium and diazinon exposure in which moreover an enlargement in the interstitial space was found (Figure 2(d)). No marked alteration in epididymal structural integrity was observed in rats after diazinon exposure (Figure 2(c)).

## 4. Discussion

Histopathology is considered the most reliable parameter for the detection of toxic effects on male reproduction [37, 38]. Therefore, macroscopic, histological and morphometric examination was carried out to determine the degree of tissue damage produced by cadmium and diazinon.

### 4.1. Cadmium Effects on Testis and Epididymis

Cadmium accumulates in male reproductive organs, in both humans and animals [39]. Numerous studies have confirmed that the testis is more sensitive to cadmium than other important organs [40, 41]. Cadmium-induced testicular toxicity is caused by the interactions between complex networks [15], involving the inhibition of oxidative stress [8], which leads to an increase in germ cell apoptosis [9] and/or distortion of the blood-testis barrier with subsequent germ cell loss, testicular edema, and hemorrhage [18, 42]. After prolonged exposure, damage inflicted by cadmium can be found at interstitial and tubular level [43]. Controversy exists regarding changes in testis weight after cadmium exposure. Some studies have reported atrophy of testis [44, 45] while others [46, 47] have reported increased testicular weight similar to the findings of the present study (Table 1). This suggests that the interstitial edema that occurred was due to fluid accumulation [37, 48]. Subchronic cadmium exposure at 30 mg/L resulted in moderate to severe testicular injury (Figure 1(b)). Regarding the tissue constituents of the testis, many irregularly outlined seminiferous tubules showed disarranged epithelial layers and necrotic cellular debris, which corroborates previous reports [43, 49]. Sloughing of cells into the lumen led to a significantly reduced percentage of the epithelial volume fraction accompanied by lumen contraction (Table 2). The formation of vacuoles in Sertoli cells and the almost complete absence of spermatozoa suggest an impairment of spermatogenesis [37]. Connective tissues were generally unaffected; nevertheless, blood vessels appeared dilated and more congested. These aforementioned observed changes agree with previous studies [16, 41] that demonstrated cadmium-induced testicular necrosis occurs after ischemia [13, 50] preceded by the perturbing of blood-testis barrier integrity and disruption of cell-to-cell endothelial and epithelial junctions [11, 12, 15].

Disruption of cell junctions and the blood-epididymis barrier has been determined as the main target of cadmium toxicity in epididymis, leading to deficient sperm maturation and motility [17, 51]. Subchronic exposure to cadmium caused epithelial thickening, and distension of tubule lumen (Table 3) together with granulomatous inflammation. Besides disruptive effects on epithelium, cadmium also induced vasoconstriction, ischemia, and edema. The edema is the direct consequence of altered hemodynamics mediated through injury to the vascular endothelium [37, 50]. Increased epididymal weight generally indicates an excessive accumulation of interstitial fluid and may be a sensitive indicator of decreased sperm production [38]. The findings are consistent with earlier reports of cadmium mediated histological changes in the testes, epididymis, and accessory sex organs [16, 52]. On the other hand, several experimental studies have presented the significant atrophy of the epididymis, decrease in the diameter of the lumen, and alkalization of the epididymis and vas deferens after cadmium administration [51, 53].

### 4.2. Diazinon Effects on Testis and Epididymis

No significant differences on the final body and testicular weight (Table 1), nor any gross pathological changes that could be attributed to diazinon exposure at 40 mg/L, were found. This biometric data is in line with what was previously reported [54]. Histopathologically, the testicular tissue showed varying degrees of distortion. Many tubules did not corroborate a significant change of germinal epithelium and interstitial tissue (Figure 1(c1)). The others appeared markedly necrotic, with degeneration of epithelial cells and only remnants of the basement membrane (Figure 1(c2)). Exfoliation of germ cells into the tubular lumen reflects the damage of Sertoli cells and the destruction of cell association [55]. Disrupting of tight junctions and adherent junctions between cells increased epithelial and endothelial permeability [42]. The morphometric analysis confirmed a significant increase of intraepithelial empty spaces accompanied by a markedly reduced tubule volume due to exposure to diazinon. In the most damaged tubules, the germ cells were not detectable, and several multinucleated cells were frequently seen together with large vacuoles. Analysis of testis sections revealed dilated and congested blood vessels (Table 2). In accordance with the present study, mild to severe degenerative changes in seminiferous tubules after exposure to various dose levels of chlorpyrifos were found [56]. Likewise, 2 weeks of exposure to diazinon resulted in a highly significant reduction of diameter size in the lumen in male adult bluegills [24]. Desquamation of germ cells, degeneration of Sertoli cells, appearance of vacuoles, and reduction in cell population occurred following exposure to quinalphos in sublethal doses [54]. Results reported herein support previous research into diazinon-induced changes to testis histopathology [25, 57] and corroborate reproductive toxicity following organophosphate exposure [58, 59]. Diazinon induces the production of oxidative stress by alteration of antioxidant enzyme activity and increasing lipid peroxidation [21]. Increased oxidative stress in the testis is associated with the suppression of Leydig cell steroidogenesis [60], disruption of spermatogenesis [29], and implications for male fertility [28].

Qualitative analysis of epididymal histology in rats exposed to diazinon showed no marked structural alterations. Compactly arranged tubules with well-organized pseudostratified epithelium and lumen filled with spermatozoa were surrounded by connective tissue without visible signs of inflammation. Absence of any significant effects after 90 days may be due to the recovery from the toxicity of diazinon [61]. Comparatively, exposure of rats to dichlorvos and diazinon for 9 weeks did not change the testis, epididymis weight, and testicular structure but the histopathological alterations were observed in the epithelial cells in epididymis, in which increased cytoplasmic vacuolation and nuclear shrinkage were found [62]. In addition, epididymis weight showed no significant change in male rats exposed to chlorpyrifos; nevertheless the epididymal tubules showed increased interstitial spaces with loss of sperm [55]. On the contrary, testis, epididymis, and prostate weight decreased significantly after exposure to diazinon for 4 weeks [25]. Malathion administration also reduced epididymal weight [63], whereas increased weight of epididymis and cellular necrosis, nuclear pyknosis, and a deleterious effect on the structural integrity following methyl parathion exposure were observed [61, 64].

### 4.3. Combined Cadmium and Diazinon Effects on Testis and Epididymis

The testicular tissue was not uniformly affected following simultaneous exposure to cadmium and diazinon. While some tubules showed developing sperm (Figure 1(d1)), others exhibited disorganization and degeneration of seminiferous epithelium, lacking the characteristic basal to luminal maturation of germ cells (Figure 1(d2)). The progressive damage was represented by seminiferous tubules devoid of germ cells and lined by Sertoli cells only [37, 41]. The weight of testes, accessory sex organs, and epididymides are the primary indicators of a possible alteration in androgen status [47]. Significantly increased testis weight after combined peroral exposure (Table 1) may reflect increased interstitial fluid and suggests injury to vascular endothelium [38]. Disruption of tight junctions perturbs endothelial barrier function and led to hemorrhaging and edema [15, 50]. Cadmium and diazinon administration caused a notable loss of spermatogenic elements. Degenerated epithelial cells were sloughed into the lumen of most seminiferous tubules. Detected desquamation of germ cells was reflected by significant vacuolisation and lumen contraction (Table 2) followed by maturation depletion [37, 54]. Furthermore, congestion and dilatation of interstitial blood vessels together with necrotizing vasculitis were identified [65]; however, observed structural alterations in testicular tissue were less expressive than after exposure to either cadmium or diazinon. The combined action of cadmium and diazinon was different from estimates based on the addition of individual responses [30]. Similarly, the coexposure to cadmium and nickel did not have a synergistic effect on testicular disturbances. Combined administration produced fewer pathological alterations than that of cadmium alone [66]. The results of the present study are similar to previous ones [67], in which a reduced effect of cadmium in combination with diazinon on bone microstructure was observed. Cadmium can affect the acetylcholinesterase enzymatic activity in a dose and duration dependent manner [68]. It was found that the activity of acetylcholinesterase decreased in short term [44] and increased after long term exposure to cadmium [69]. Therefore, it is possible that activation of acetylcholinesterase followed after prolonged cadmium administration because of similarity to calcium, which increases enzymatic activity [70]. In the approach used in this study, a possible mechanism of action of combined exposure to cadmium and diazinon was not modeled, but rather we assumed that the reduced toxicity was due to competitive interactions among chemicals [71].

Histological examination did not reveal differences in epididymal morphology of rats, except the cytoplasmatic vacuolisation within epithelium following simultaneous exposure [37, 38]. Nevertheless, the morphometric analysis revealed increased epididymis weight and epithelial thickening accompanied by widening of the interstitial space (Table 3). Similarly, the dose-dependent epididymal toxicity induced by dichlorvos, dimethoate, and malathion mixture was associated with alterations on epididymis weight, structure, and function [72]. Reduction in capillary size confirms that cadmium-induced testicular and epididymal pathophysiology is mediated by perturbing the vascular system [50]. Any structural alteration may adversely affect function of the epididymal epithelium, which could impact sperm maturation [17]. Heavy metal and pesticide exposure are potential risk factors for adverse reproductive health outcomes, including poor semen quality [1, 3, 73].

## 5. Conclusion

The aim of the present study was to assess the impact of cadmium and diazinon on the rat testis and epididymis by using various qualitative and quantitative histological methods. Effects on the testis included vascular disruption, interstitial edema and hemorrhage, germ cell loss, cellular degeneration, and necrosis with epithelial vacuolisation. However, observed degenerative changes after simultaneous exposure were significantly less expressive as compared to exposure to the individual compounds. Epithelial thickening, necrotic epithelial cells, vasoconstriction, interstitial edema, and mononuclear cell infiltration were most detectable in epididymis following exposure to cadmium either alone or in combination. In summary, cadmium and diazinon induced significant structural alterations in testicular and epididymal tissue which may affect male reproduction. The underlying molecular mechanism of the interaction of cadmium with diazinon should be explored in future studies.

## Figures and Tables

**Table 1:** Biometric parameters of control and experimental rat males.

Parameter	Group A	Group B	Group C	Group D
Final body weight (g)	405.00 ± 52.65	426.67 ± 25.25	406.00 ± 24.70	427.78 ± 19.22
Testis weight (g)	1.50 ± 0.17	1.68 ± 0.10^*^	1.36 ± 0.24	1.75 ± 0.18^**^
Epididymis weight (g)	0.55 ± 0.05	0.65 ± 0.07^**^	0.55 ± 0.10	0.66 ± 0.06^***^

The data is presented as mean and standard deviation; ^*^ *P* < 0.05; ^**^ *P* < 0.01; ^***^ *P* < 0.001.

**Table 2:** Morphometric testicular parameters.

Parameter	Group A	Group B	Group C	Group D
Seminiferous epithelium (%)	64.82 ± 2.90	53.67 ± 4.31^***^	67.73 ± 4.81	65.76 ± 2.89
Seminiferous epithelium (*µ*m^2^)	918.82 ± 41.06	760.75 ± 61.07^***^	960.05 ± 68.18	932.09 ± 41.02
Intraepithelial spaces (%)	0.15 ± 0.18	12.79 ± 3.93^***^	5.09 ± 1.46^***^	2.96 ± 2.04^***^
Intraepithelial spaces (*µ*m^2^)	2.17 ± 2.57	181.25 ± 55.72^***^	72.11 ± 20.63^***^	42.02 ± 28.96^***^
Tubule lumen (%)	23.58 ± 3.03	19.75 ± 4.14^*^	14.24 ± 3.51^***^	18.26 ± 4.45^**^
Tubule lumen (*µ*m^2^)	334.29 ± 42.91	279.99 ± 58.69^*^	201.86 ± 49.81^***^	258.79 ± 63.02^**^
Interstitial tissue (%)	10.93 ± 2.03	11.74 ± 3.06	11.77 ± 1.70	12.01 ± 1.43
Interstitial tissue (*µ*m^2^)	154.86 ± 28.71	166.46 ± 43.37	166.89 ± 24.14	170.22 ± 20.30
Blood vessels (%)	0.29 ± 0.27	2.05 ± 1.36^***^	1.17 ± 0.51^***^	1.01 ± 0.56^**^
Blood vessels (*µ*m^2^)	4.16 ± 3.77	29.05 ± 19.34^***^	16.61 ± 7.25^***^	14.38 ± 7.96^**^
Tubule surface (*µ*m^2^)	372.19 ± 69.04	367.87 ± 41.14	364.57 ± 26.17	360.21 ± 32.99
Tubule diameter (*µ*m^2^)	276.22 ± 26.28	281.67 ± 19.58	273.98 ± 9.90	272.55 ± 12.41

The data is presented as mean and standard deviation; ^*^ *P* < 0.05; ^**^ *P* < 0.01; ^***^ *P* < 0.001.

**Table 3:** Morphometric epididymal parameters.

Parameter	Group A	Group B	Group C	Group D
Epididymal epithelium (%)	21.86 ± 2.91	18.65 ± 1.19^**^	20.12 ± 4.58	18.75 ± 3.32^*^
Epididymal epithelium (*µ*m^2^)	309.92 ± 41.31	264.31 ± 16.85^**^	285.21 ± 64.91	265.81 ± 47.04^*^
Tubule lumen (%)	69.37 ± 4.79	73.74 ± 1.61^*^	69.05 ± 7.07	67.25 ± 4.06
Tubule lumen (*µ*m^2^)	983.35 ± 67.91	1045.21 ± 22.78^*^	978.76 ± 100.16	953.23 ± 57.59
Interstitial tissue (%)	8.29 ± 2.10	7.46 ± 1.20	10.37 ± 3.17	13.87 ± 2.54^***^
Interstitial tissue (*µ*m^2^)	117.50 ± 29.80	105.75 ± 17.05	146.99 ± 44.90	196.62 ± 36.07^***^
Blood vessels (%)	0.29 ± 0.15	0.16 ± 0.11^*^	0.26 ± 0.15	0.13 ± 0.12^*^
Blood vessels (*µ*m^2^)	4.14 ± 2.14	2.23 ± 1.54^*^	3.70 ± 2.16	1.87 ± 1.68^*^
Tubule surface (*µ*m^2^)	314.32 ± 13.10	335.19 ± 23.43^*^	318.19 ± 25.47	336.05 ± 48.01
Tubule diameter (*µ*m^2^)	254.64 ± 5.30	262.87 ± 9.18^*^	256.07 ± 10.28	262.71 ± 19.32

The data is presented as mean and standard deviation; ^*^ *P* < 0.05; ^**^ *P* < 0.01; ^***^ *P* < 0.001.
